# Trends and urban–rural disparities in infant and cause-specific mortality in Shaanxi Province, China, 2014–2023: a population-based study

**DOI:** 10.3389/fpubh.2026.1801740

**Published:** 2026-04-14

**Authors:** Xue Yang, Baozhu Wang, Juan Zhang

**Affiliations:** 1 Department of Health, Northwest Women's and Children's Hospital, Xi'an, Shaanxi, China; 2 Department of Neonatology, Northwest Women's and Children's Hospital, Xi'an, Shaanxi, China

**Keywords:** accidental asphyxia, cause-specific mortality, congenital anomalies, infant mortality, Shaanxi province, urban–rural disparities

## Abstract

**Background:**

China has achieved substantial reductions in infant mortality; however, pronounced subnational disparities persist, particularly in western and rural regions. In the context of demographic change and evolving perinatal risk profiles, region-specific evidence is essential to guide targeted interventions and support progress toward Sustainable Development Goal (SDG) 3.2.

**Methods:**

This population-based observational study analyzed data from the Maternal and Child Health Surveillance System (MCHSS) in Shaanxi Province, China, from 2014 to 2023. A total of 320,259 live births and 954 infant deaths were recorded across 141 surveillance sites. Temporal trends in infant mortality rates (IMRs) were assessed using Joinpoint regression to estimate average annual percentage changes (AAPCs). Urban–rural differences and temporal trends were further evaluated using generalized linear models.

**Results:**

The IMR in Shaanxi Province declined from 4.18 per 1,000 live births in 2014 to 2.16 in 2023 (AAPC: −6.31%, *P* < 0.001). Declines were observed in rural (AAPC: −10.58%, *P* < 0.001) and urban areas (AAPC: −5.44%, *P* = 0.003), although regression analysis did not identify statistically significant differences in temporal trends between residence groups (*P* = 0.152). Most infant deaths occurred during the neonatal period (62.1%). The leading causes of infant death were congenital heart defects (CHDs), birth asphyxia, pre-term birth and/or low birth weight (preterm/LBW), pneumonia, other congenital anomalies, and accidental asphyxia. Cause-specific mortality from congenital anomalies declined significantly (CHDs: AAPC −6.18%, *P* = 0.002; other congenital anomalies: AAPC −6.44%, *P* = 0.002). Pneumonia-related mortality also decreased markedly, particularly in urban areas (AAPC: −41.19%, *P* = 0.028).

**Conclusion:**

Infant mortality in Shaanxi Province declined substantially between 2014 and 2023, with broadly parallel trends in urban and rural populations. Despite this progress, pre-maturity-related conditions and injuries remain important contributors to infant death. Strengthening neonatal care systems, expanding birth defect prevention programs, and implementing targeted injury prevention strategies, particularly in underserved rural areas, may support further reductions in infant mortality and promote equitable progress toward SDG 3.2.

## Introduction

Over the past few decades, China has made remarkable strides in reducing infant mortality, successfully meeting key targets outlined in Millennium Development Goal (MDG) 4 (1). Between 1990 and 2020, the national infant mortality rate (IMR) dropped substantially, from 50.2 to 5.4 deaths per 1,000 live births, representing one of the most rapid declines globally (2, 3).

Despite this overall national success, pronounced rural–urban disparities in infant mortality persist, and in some regions, particularly in western provinces such as Shaanxi, these disparities may have widened over time (4). Both overall IMRs and cause-specific mortality patterns vary considerably across subnational contexts. Such disparities are further shaped by China's ongoing demographic transition. Following the implementation of the three-child policy in 2021, the proportion of births to women of advanced maternal age has increased, a well-established risk factor for pre-term birth and severe neonatal morbidity (5).

In the post-MDG era, Sustainable Development Goal (SDG) 3.2 and China's Children's Development Outline (2021–2030) aim to eliminate preventable deaths among children under five by 2030, with an explicit focus on reducing health inequities (6, 7). However, much of the existing literature relies on nationally aggregated data, which masks important subnational variations and limits the ability to identify locally relevant risk patterns (1). The lack of region-specific evidence constrains the development of targeted public health interventions, especially in socioeconomically diverse and resource-constrained settings.

To address this evidence gap, we analyzed population-based data from the Maternal and Child Health Surveillance System (MCHSS) in Shaanxi Province between 2014 and 2023. This study investigates temporal trends in overall and cause-specific infant mortality, evaluates urban–rural disparities, and identifies emerging risk patterns at the subprovincial level. By delineating high-risk regions, vulnerable age groups, and leading causes of death, the findings aim to support evidence-based policymaking and guide the design of targeted child health strategies. Beyond its relevance for western China, the study may offers insights that inform efforts in other low- and middle-income countries working toward the achievement of SDG targets.

## Materials and methods

### Study design and population

This population-based observational study was conducted using data from the Chinese National MCHSS in Shaanxi Province between January 1, 2014, and December 31, 2023. According to the regional classification criteria of the National Development and Reform Commission of China, Shaanxi Province is administratively divided into eastern, central, and western regions, each further categorized into urban and rural areas.

Under the national surveillance framework, a stratified design was implemented to enhance geographic and socioeconomic representation. The province was first stratified by region (eastern, central, and western), and within each stratum, both urban and rural areas were included to capture differences in population distribution and health service capacity. Selected districts and counties were designated as surveillance areas in accordance with national guidelines. Within these designated areas, all townships in rural settings and all communities in urban settings were incorporated as monitoring units rather than being individually sampled.

Across the province's 10 prefecture-level cities, 141 townships or urban communities located in 6 cities and 9 districts/counties were included in the national maternal and child health surveillance network. During the study period, approximately 0.32 million live births were recorded within these surveillance areas, accounting for about 9% of all live births in Shaanxi Province. The male-to-female sex ratio at birth was 107.7 in the surveillance sites, compared with 108.7 at the provincial level (8). The close similarity between these estimates supports the demographic comparability of the surveillance population with the broader provincial population and the suitability of the dataset for provincial-level trend analyses.

The study population included all children under 5 years of age who were permanent residents of the surveillance sites or had resided in the catchment area for at least 1 year. All live births to mothers who were registered residents of the surveillance sites or had lived in the area for at least 1 year were included. Stillbirths, defined as fetuses showing no signs of life during labor or delivery, were excluded. Detailed descriptions of MCHSS coverage, sampling, and operational procedures have been reported elsewhere (1).

This study utilized de-identified data extracted from the MCHSS, with a waiver of informed consent granted in accordance with applicable regulations (1). The study was conducted in adherence to the Strengthening the Reporting of Observational Studies in Epidemiology guidelines.

### Data collection and quality control

Within each surveillance site, live births and child deaths were initially recorded by village doctors or community physicians and subsequently reported to township health centers or community health service centers. Live birth data were reported quarterly, whereas child deaths were reported within 3 days of occurrence. After preliminary verification, standardized child death report cards were completed within 7 days and submitted through a hierarchical reporting system to district/county, prefectural, provincial, and national maternal and child health institutions.

For each reported child death, trained health-care workers conducted household investigations to collect detailed information, including date of birth, sex, gestational age, place of death, clinical diagnosis, health-care utilization, and treatments received prior to death. Causes of death were determined by panels of clinical specialists based on available medical records and diagnostic evidence. For deaths occurring outside health facilities, causes of death were assigned using information obtained from caregiver interviews in accordance with the World Health Organization (WHO) 2016 Verbal Autopsy Standards (9).

All mortality data were entered into a web-based reporting system and underwent multi-tier verification procedures. These included monthly reviews of death reports at township-level facilities, quarterly supervisory visits at the district and county levels, and annual quality assessments conducted by prefectural, provincial, and national authorities. To further ensure data completeness, multi-source data cross-validation was conducted in selected surveillance areas by linking records with hospitals, Centers for Disease Control and Prevention, Public Security Bureaus, and Civil Affairs Bureaus. Records with implausible gestational age or birth weight values were verified against original source documents. Cases with missing key variables (< 1%) were excluded from variable-specific analyses but retained for overall mortality calculations. Comprehensive descriptions of the data collection and quality control procedures have been reported elsewhere (10).

### Variable definitions

A live birth was defined as the delivery of a fetus at or beyond 28 completed weeks of gestation, or with a birth weight greater than 1,000 g, exhibiting at least one sign of life, such as a heartbeat, breathing, umbilical cord pulsation, or voluntary muscle movement. This definition follows the national reporting standards used in the Chinese MCHSS. The neonatal mortality rate (NMR) and IMR were calculated as period measures. The NMR was defined as the number of neonatal deaths (from birth to 27 completed days of life) occurring in calendar year *t* divided by the number of live births in calendar year *t*, expressed per 1,000 live births. The IMR was defined as the number of infant deaths (before 1 year of age) occurring in calendar year *t* divided by the number of live births in calendar year *t*, expressed per 1,000 live births. Low birth weight (LBW) was defined as a birth weight of less than 2,500 g. Pre-term birth was defined as delivery before 37 completed weeks of gestation. For descriptive analyses, pre-term births were further categorized as extremely pre-mature (< 28 completed weeks of gestation) and moderate pre-term (28–36 completed weeks of gestation). During the study period, births at < 28 weeks accounted for approximately 0.01% of all recorded live births, suggesting a minimal influence on provincial infant mortality estimates.

Causes of death were classified using the International Classification of Diseases, Tenth Revision (11). “Other congenital anomalies” included all congenital defects, such as neural tube defects, Down syndrome, cleft lip and palate, limb deformities, and esophageal atresia, excluding congenital heart defects (CHDs). For analytical purposes, causes of death other than CHDs, birth asphyxia, pre-term birth and/or low birth weight (preterm/LBW), pneumonia, other congenital anomalies, and accidental asphyxia were grouped as “other causes”. These six leading causes collectively accounted for 75.1% (*n* = 716) of all infant deaths.

### Statistical analysis

Data from the MCHSS covering Shaanxi Province for the period 2014–2023 were extracted and compiled into a final analytical dataset using Microsoft Excel 2016. Range and logical consistency checks were performed to identify and correct any data inconsistencies.

Continuous variables were summarized as means and standard deviations, while categorical variables were presented as frequencies and percentages. Bar charts were used to visualize the distribution of infant death causes. Pearson's chi-squared tests were applied to compare categorical variables. Independent-samples *t*-tests were used to compare continuous variables between urban and rural populations.

To formally evaluate temporal trends and urban–rural differences in IMRs, generalized linear models were fitted using annual infant death counts as the dependent variable and the number of live births as an offset term (log scale). A log link function was specified. In multivariable Poisson regression models, calendar year was included as a continuous variable to assess linear temporal trends. Urban–rural residence was entered as a binary indicator variable. To formally test whether mortality trends differed by residence, an interaction term between calendar year and residence was included. Rate ratios (RRs) and 95% confidence intervals (CIs) were estimated from model coefficients.

Temporal trends in IMR and cause-specific mortality rates were additionally described using Joinpoint regression (Joinpoint Regression Program, version 5.3.0; National Cancer Institute) (12). Joinpoint models were fitted on the log-transformed rates, allowing a maximum of one joinpoint given the limited number of annual observations (*n* = 10). Model selection was guided by the Bayesian Information Criterion, and statistical significance was determined using the Monte Carlo permutation method. Average annual percentage changes (AAPCs) and corresponding 95% CIs were reported. An AAPC greater than zero indicated an increasing trend, while an AAPC less than zero indicated a decreasing trend.

All statistical analyses were performed using SPSS software (version 22.0; SPSS Inc., Chicago, IL, USA). The study adhered to relevant guidelines and regulations throughout.

## Results

### Demographic characteristics

Between 2014 and 2023, a total of 320,259 live births and 954 infant deaths were reported across the Maternal and Child Health Surveillance sites in Shaanxi Province. Among infant deaths, 548 occurred in males (57.4%) and 406 in females (42.6%). Of all deaths among children under 5 years of age (*n* = 1,331), infant deaths accounted for 71.7% (954/1,331), while neonatal deaths comprised 44.5% (592/1,331).

Most infant deaths occurred during the neonatal period, accounting for 60.3% (117/194) of urban and 62.5% (475/760) of rural infant deaths. The mean gestational age of deceased infants was significantly lower in urban areas than in rural areas (36.3 ± 4.2 vs. 37.1 ± 3.9 weeks, *P* = 0.013). Conversely, the mean birth weight was slightly higher in rural areas than in urban areas (2,702.9 ± 821.8 g vs. 2,571.3 ± 832.5 g, *P* = 0.050).

A significantly higher proportion of urban infants who died received a definitive diagnosis at provincial- or municipal-level medical institutions compared with rural infants (78.4 vs. 62.2%, *P* < 0.001). Extremely pre-term deaths accounted for 3.6% of urban infant deaths and 2.1% of rural infant deaths, with no statistically significant difference between settings (*P* = 0.142; Table 1).

**Table 1:** Demographic and clinical characteristics of infant deaths in urban and rural areas of Shaanxi Province, 2014–2023.

Variable	Urban	Rural	T/χ^2^	*p*-value
Gestation age at birth (weeks), (mean ± Sd)	36.3 ± 4.2	37.1 ± 3.9	2.500	0.013
Birth weight (g), (mean ± Sd)	2,571.3 ± 832.5	2,702.9 ± 821.8	1.960	0.050
Sex, *n* (%)				
Male	112 (57.7)	436 (57.4)	0.008	0.927
Female	82 (42.3)	324 (42.6)		
Preterm categories, *n* (%)				
Extremely pre-mature (< 28 week)	7 (3.6)	16 (2.1)	3.907	0.142
Moderate pre-mature (28–36 week)	60 (30.9)	199 (26.2)		
Term (≥37 week)	124 (63.9)	541 (71.2)		
Birth place, *n* (%)				
Provincial/municipal hospital	134 (69.1)	263 (34.6)	76.355	< 0.001
District/township hospital	58 (29.9)	484 (63.7)		
Way to hospital	1 (0.5)	11 (1.4)		
Neonate diagnosis facilities level, *n* (%)				
Provincial/municipal level	152 (78.4)	473 (62.2)	18.150	< 0.001
District/county level	29 (14.9)	207 (27.2)		
Street/village level	1 (0.1)	11 (1.4)		
Have not seek medical advises	12 (6.2)	69 (9.1)		
Death age, *n* (%)				
< 7 days	91 (46.9)	365 (48.0)	0.360	0.835
7–27 days	26 (13.4)	110 (14.5)		
≥28 days	77 (39.7)	285 (37.5)		
Death place, *n* (%)				
Hospital	102 (52.6)	379 (49.9)	0.661	0.718
Way to hospital	30 (15.5)	134 (17.6)		
Home	62 (32.0)	247 (32.5)		
Death season, *n* (%)				
Spring	52 (26.8)	182 (23.9)	6.412	0.093
Summer	38 (19.6)	158 (20.8)		
Autumn	40 (20.6)	217 (28.6)		
Winter	64 (33.0)	203 (26.7)		
Causes of death, *n* (%)				
Congenital heart defects	21 (10.8)	132 (17.4)	18.854	0.004
Birth asphyxia	28 (14.4)	119 (15.7)		
Preterm/LBW	42 (21.6)	93 (12.2)		
Pneumonia	22 (11.3)	79 (10.4)		
Other congenital anomalies	12 (6.2)	84 (11.1)		
Accidental asphyxia	21 (10.8)	63 (8.3)		
Others	48 (24.7)	190 (25.0)		

LBW, low birth weight; SD, standard deviation.

Overall, the six leading causes of infant death were CHDs (*n* = 153, 16.0%), birth asphyxia (*n* = 147, 15.4%), pre-term/LBW (*n* = 135, 14.2%), pneumonia (*n* = 101, 10.6%), other congenital anomalies (*n* = 96, 10.1%), and accidental asphyxia (*n* = 84, 8.8%). Birth asphyxia-related deaths were pre-dominantly concentrated in the early neonatal period (95.2%, 140/147), whereas deaths due to CHDs and pneumonia occurred mainly in the late neonatal period (73.9 and 76.2%, respectively; *P* < 0.001; Supplementary Table S1).

### Trends in infant mortality rates (2014–2023)

**Figure 1:**
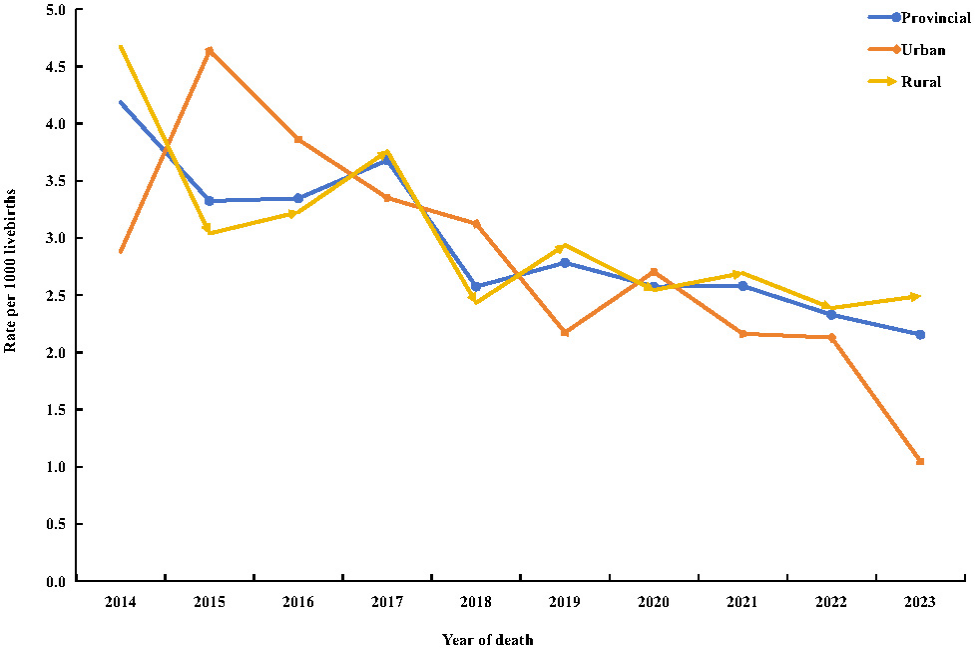
Trends in infant mortality rate in Shaanxi Province, 2014–2023.

Between 2014 and 2023, IMRs in Shaanxi Province declined steadily (Figure 1). At the provincial level, the IMR decreased from 4.18 per 1,000 live births (95% CI: 2.74–5.63) in 2014 to 2.16 per 1,000 live births (95% CI: 1.52–2.80) in 2023. Using 2014 as the reference year, the RR in 2023 was 0.515 (95% CI: 0.327–0.812; *P* = 0.004), indicating a statistically significant decline over time. Joinpoint regression analysis further demonstrated a significant AAPC of −6.31% (95% CI: −8.17 to −4.47%; *P* < 0.001) at the provincial level.

Similar declining trends were observed in both rural and urban populations. In rural areas, the IMR decreased from 4.67 (95% CI: 2.88–6.46) in 2014 to 2.49 (95% CI: 1.71–3.28) in 2023 (RR: 0.534; 95% CI: 0.325–0.877; *P* = 0.013), with a significant AAPC of −10.58% (95% CI: −15.55 to −5.33%; *P* < 0.001). In urban areas, the IMR decreased from 2.88 (95% CI: 0.58–5.19) in 2014 to 1.05 (95% CI: 0.13–1.97) in 2023; however, due to smaller case numbers and wide confidence intervals, this reduction did not reach statistical significance in 2023 (RR: 0.364; 95% CI: 0.111–1.192; *P* = 0.095; Table 2). Joinpoint analysis indicated a significant downward trend in urban areas, with an AAPC of −5.44% (95% CI: −8.88 to −1.97%; *P* = 0.003).

**Table 2:** Trend of infant mortality rate (IMR, per 1,000 live births) in Shaanxi Province, 2014–2023.

Year	Provincial			Urban			Rural		
	IMR (95% CI)	RR (95% CI)	*p*-value	IMR (95% CI)	RR (95% CI)	*p*-value	IMR (95% CI)	RR (95% CI)	*p*-value
2014	4.18 (2.74, 5.63)	1.000	—	2.88 (0.58, 5.19)	1.000	—	4.67 (2.88, 6.46)	1.000	—
2015	3.32 (2.78, 3.87)	0.794 (0.514, 1.166)	0.240	4.64 (3.10, 6.17)	1.609 (0.677, 3.824)	0.282	3.04 (2.46, 3.62)	0.651 (0.424, 0.999)	0.050
2016	3.35 (2.81, 3.88)	0.800 (0.546, 1.171)	0.251	3.86 (2.55, 5.18)	1.340 (0.561, 3.197)	0.510	3.23 (2.64, 3.81)	0.691 (0.452, 1.056)	0.087
2017	3.68 (3.13, 4.23)	0.879 (0.603, 1.283)	0.505	3.35 (2.15, 4.55)	1.162 (0.484, 2.793)	0.736	3.76 (3.14, 4.38)	0.804 (0.529, 1.222)	0.308
2018	2.57 (2.06, 3.09)	0.615 (0.413, 0.918)	0.017	3.12 (1.88, 4.37)	1.084 (0.443, 2.652)	0.859	2.43 (1.88, 2.99)	0.521 (0.333, 0.815)	0.004
2019	2.78 (2.26, 3.31)	0.665 (0.448, 0.987)	0.043	2.17 (1.14, 3.21)	0.754 (0.297, 1.913)	0.552	2.94 (2.34, 3.54)	0.629 (0.407, 0.972)	0.037
2020	2.58 (2.03, 3.13)	0.616 (0.410, 0.925)	0.020	2.70 (1.42, 3.99)	0.938 (0.370, 2.380)	0.894	2.54 (1.94, 3.15)	0.545 (0.346, 0.857)	0.009
2021	2.58 (1.97, 3.19)	0.617 (0.405, 0.939)	0.024	2.16 (0.94, 3.39)	0.751 (0.282, 2.000)	0.566	2.69 (1.99, 3.40)	0.576 (0.362, 0.918)	0.020
2022	2.33 (1.70, 3.00)	0.557 (0.359, 0.863)	0.009	2.13 (0.87, 3.39)	0.739 (0.273, 1.998)	0.551	2.39 (1.67, 3.11)	0.511 (0.313, 0.833)	0.007
2023	2.16 (1.52, 2.80)	0.515 (0.327, 0.812)	0.004	1.05 (0.13, 1.97)	0.364 (0.111, 1.192)	0.095	2.49 (1.71, 3.28)	0.534 (0.325, 0.877)	0.013

RR, rate ratio; CI, confidence interval.

In the multivariable Poisson regression analysis (Lagrange multiplier test, *P* = 0.229; Pearson χ^2^ goodness-of-fit test, *P* = 0.997), calendar year was significantly associated with infant mortality (*P* < 0.001), indicating a significant downward temporal trend. Urban–rural residence was not independently associated with IMR after adjustment for calendar year (*P* = 0.983), and the interaction between calendar year and residence was not statistically significant (*P* = 0.152; Table 2).

### Cause-specific mortality patterns

**Figure 2:**
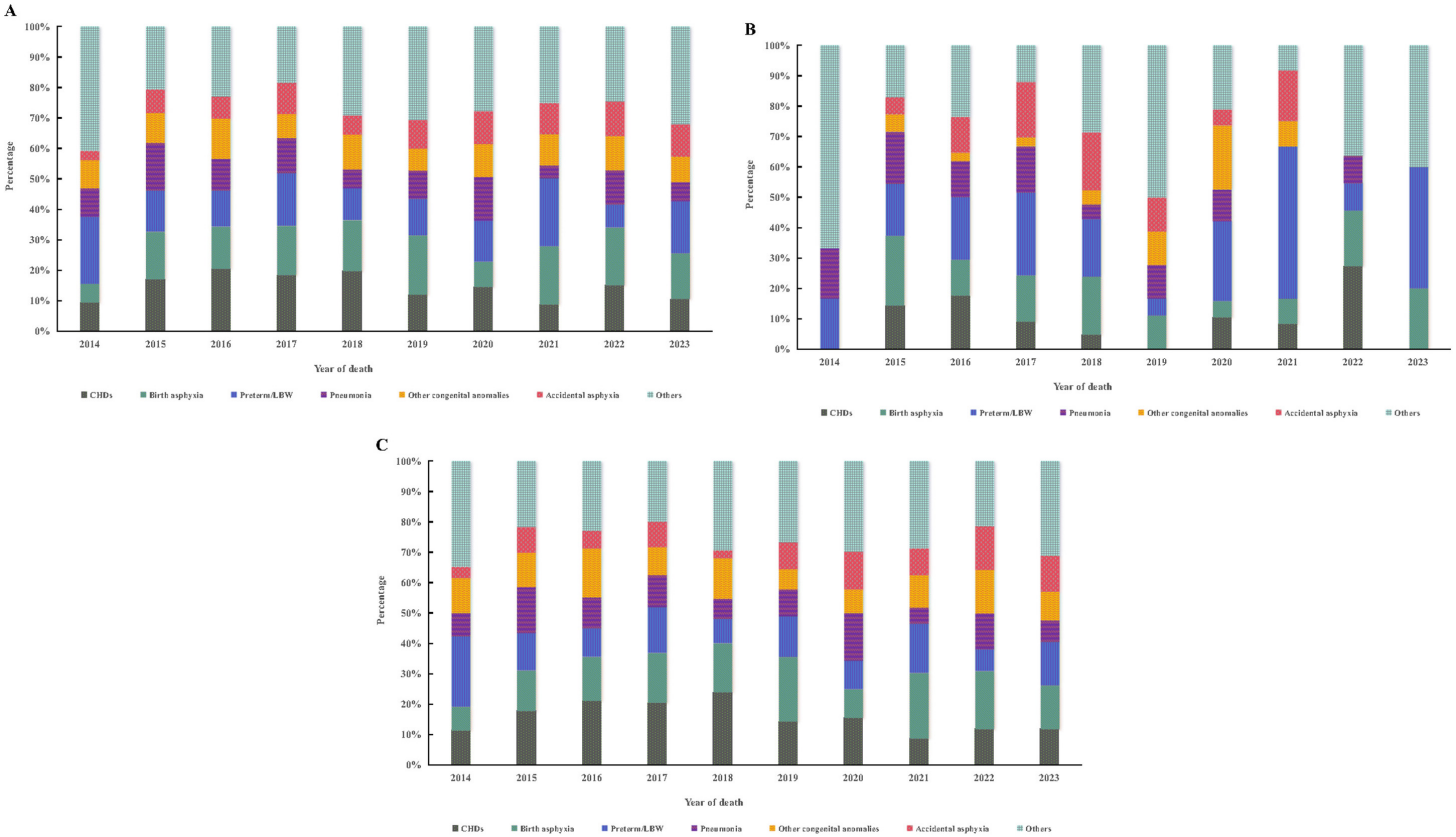
Distribution of causes of infant death in Shaanxi Province, 2014–2023. **(A)** Overall provincial distribution; **(B)** Urban areas; **(C)** Rural areas. CHDs, congenital heart defects; LBW, low birth weight.

Throughout the study period, CHDs, birth asphyxia, and pre-term/LBW consistently ranked as the three leading causes of infant death, collectively accounting for approximately 40% of all deaths. When examining the composition among infant deaths, the proportion attributable to pneumonia declined significantly over time (*P* < 0.001), whereas the proportion attributable to pre-term/LBW increased (*P* < 0.001; Figure 2).

At the provincial level, cause-specific mortality rates due to CHDs (AAPC: −6.18%, *P* = 0.002) and other congenital anomalies (AAPC: −6.44%, *P* = 0.002) declined significantly during the study period. Mortality attributable to other congenital anomalies showed the largest average annual decline. In contrast, mortality due to accidental asphyxia increased from 0.13 to 0.25 per 1,000 live births between 2014 and 2023; however, this upward trend was not statistically significant (*P* = 0.331; Table 3).

**Table 3:** Leading causes of infant death in Shaanxi Province, 2014–2023.

Causes of death	Cause-specific mortality (per 100,000 livebirths)										AAPC [% (95% CI)]	*p*-value
	2014	2015	2016	2017	2018	2019	2020	2021	2022	2023		
Provincial												
Congenital heart defects	39.23	56.57	68.70	68.86	50.44	33.51	36.79	22.77	35.15	24.49	−6.18 (−11.25, −2.02)	0.002
Birth asphyxia	26.15	51.86	46.54	60.25	42.48	54.13	21.46	49.35	43.94	34.29	−0.78 (−8.05, 6.90)	0.843
Preterm/LBW	91.54	44.79	39.89	64.56	26.55	33.51	33.73	56.94	17.58	39.19	−8.20 (−25.18, 12.06)	0.310
Pneumonia	39.23	51.86	35.46	43.04	15.93	25.78	36.79	11.39	26.36	14.70	−11.01 (−22.20, 1.49)	0.079
Other congenital anomalies	39.23	33.00	44.32	30.13	29.20	20.62	27.60	26.57	26.36	19.59	−6.44 (−10.49, −2.29)	0.002
Accidental asphyxia	13.08	25.93	24.38	38.73	15.93	25.78	27.60	26.57	26.36	24.49	3.49 (−3.26, 10.35)	0.331
Others	170.00	68.36	77.56	68.86	74.33	85.06	70.52	64.53	57.12	73.48	−7.81 (−11.38, −2.08)	< 0.001
Urban area												
Congenital heart defects	0.00	66.23	70.18	33.49	13.02	0.00	31.81	18.02	58.07	0.00	−11.39 (−51.48, 61.87)	0.692
Birth asphyxia	0.00	105.96	46.79	55.82	52.10	25.57	15.90	18.02	38.71	20.96	30.11 (−10.7, 67.42)	0.162
Preterm/LBW	48.03	79.47	81.88	100.47	52.10	12.78	79.52	108.15	19.36	41.92	−6.92 (−23.01, 12.18)	0.432
Pneumonia	48.03	79.47	46.79	55.82	13.02	25.57	31.81	0.00	19.36	0.00	−41.19 (−64.18, −5.02)	0.028
Other congenital anomalies	0.00	26.49	11.70	11.16	13.02	25.57	63.61	18.02	0.00	0.00	−29.54 (−53.06, 2.96)	0.068
Accidental asphyxia	0.00	26.49	46.79	66.98	52.10	25.57	15.90	36.05	0.00	0.00	−13.12 (−51.13, 47.71)	0.425
Other diseases	192.12	79.47	93.58	44.65	78.15	115.05	63.61	18.02	77.43	41.92	−11.77 (−26.13, 5.09)	0.152
Rural area												
Congenital heart defects	53.91	54.48	68.35	77.30	60.02	41.97	37.99	24.04	28.42	31.96	−8.54 (−13.61, −3.78)	< 0.001
Birth asphyxia	35.94	40.15	46.48	61.31	40.01	61.34	22.79	57.70	45.48	38.36	−0.001 (−7.68, 8.56)	0.981
Preterm/LBW	107.82	37.28	30.07	55.98	20.01	38.74	22.79	43.28	17.05	38.36	−8.69 (−24.09, 9.77)	0.273
Pneumonia	35.94	45.88	32.81	39.99	16.67	25.83	37.99	14.43	28.42	19.18	−7.48 (−18.51, 5.17)	0.198
Other congenital anomalies	53.91	34.41	51.95	34.65	33.34	19.37	18.99	28.85	34.11	25.57	−5.66 (−14.61, 2.27)	0.125
Accidental asphyxia	17.97	25.81	19.14	31.99	6.67	25.83	30.39	24.04	34.11	31.96	5.91 (−8.00, 21.94)	0.414
Others	161.73	65.96	73.82	74.64	73.36	77.48	72.17	76.93	51.16	83.10	−4.51 (−11.45, 2.72)	0.200

AAPC, average annual percentage change; CI, confidence interval; LBW, low birth weight.

A pronounced reduction was also observed in pneumonia-related mortality in urban areas (AAPC: −41.2%, *P* = 0.028), exceeding the provincial average decline. In rural areas, CHD-related mortality demonstrated a significant downward trend (AAPC: −8.5%, *P* < 0.001).

## Discussion

This population-based study documented a substantial and sustained decline in infant mortality in Shaanxi Province between 2014 and 2023. The IMR decreased at an average annual rate of 6.31%, reaching 2.16 deaths per 1,000 live births in 2023. This level is lower than the national average reported for the same period and meets the target set by the China Children's Development Outline (2021–2030) ahead of schedule (7). In an international context, Shaanxi's IMR was also considerably lower than those reported in several upper-middle-income countries, including Brazil, South Africa, Mexico, and Malaysia, placing the province among regions with comparatively low infant mortality levels (3). Notably, even in rural areas, traditionally characterized by limited access to health-care services, the IMR remained well below the SDG 3.2 threshold of 12 deaths per 1,000 live births (6).

Declines in IMR were observed in both urban and rural populations. Although descriptive analyses suggested a numerically steeper decline in rural areas, formal regression-based interaction testing did not identify statistically significant differences in temporal trends between residence groups in the study. These findings support broadly parallel downward trajectories in urban and rural settings rather than evidence of differential rates of decline (13). The observed reductions in IMR also occurred during a period of continued strengthening of national maternal and child health initiatives, including the Healthy Children Action Enhancement Plan and the Maternal and Infant Safety Action Plan (1, 7). Although causal relationships cannot be established in this ecological analysis, the temporal alignment between policy implementation and declining mortality may suggest that expanded service coverage, improved referral systems, and enhanced perinatal care capacity contributed to sustained progress.

Despite overall improvements, structural disparities in health system capacity remain evident. A substantially higher proportion of infant deaths in urban areas occurred in tertiary-level medical institutions compared with rural areas, reflecting differential access to advanced diagnostic and treatment services. This distribution may indicate stronger referral systems and greater availability of specialized neonatal care in urban settings. In contrast, geographic barriers and limited infrastructure in rural areas may continue to constrain timely access to high-level care (14). Continued investment in rural health systems, including strengthening referral networks, enhancing county-level hospital capacity, and supporting workforce retention, will be essential to sustain and further advance reductions in infant mortality.

Consistent with global patterns (14–16), more than 60% of infant deaths occurred during the neonatal period, underscoring the persistent vulnerability of early life even in settings with relatively low overall mortality. Improving neonatal outcomes has therefore remained a strategic priority for child health in the province. By 2023, a network of 133 neonatal critical care centers had been established, achieving full coverage across provincial, municipal, and county levels (17). The expansion of neonatal intensive care capacity may reflect sustained efforts to improve the survival prospects of high-risk newborns. Nevertheless, continued emphasis on early risk identification, timely referral, and high-quality intrapartum and immediate neonatal care remains essential for further reducing neonatal mortality.

With respect to causes of death, cause-specific mortality rates declined for several major conditions, including CHDs and other congenital anomalies. The reduction in mortality attributable to congenital anomalies may be associated with expanded prenatal screening, early diagnosis, and improved neonatal management (17, 18). Similar patterns have been reported in other settings following strengthening of birth defect prevention and perinatal care systems (19, 20). Although the composition among infant deaths attributable to pneumonia decreased over time, pneumonia-related mortality rates also declined substantially, particularly in urban areas. This pattern is consistent with trends reported both nationally and internationally, where improvements in infection control and access to basic health services have contributed to reductions in infectious mortality (1, 21–23).

Birth asphyxia and pre-maturity remain major contributors to infant mortality in both urban and rural settings. The persistence of mortality from these conditions may reflect the increasing clinical complexity of residual cases as overall mortality declines (10, 11). Evidence-based interventions, including intrauterine transfer for high-risk pregnancies, kangaroo mother care, and standardized neonatal resuscitation protocols, remain essential components of strategies aimed at further reducing neonatal and infant mortality.

As mortality from infectious diseases declines, non-communicable perinatal conditions and injury-related causes account for a greater proportion of infant deaths. This shift reflects changes in the composition among infant deaths rather than clear increases in cause-specific mortality rates. Although mortality from accidental asphyxia did not show a statistically significant increase during the study period, its relatively stable level underscores the continuing importance of injury prevention (24). National surveillance data also indicate that the proportion of injury-related deaths among children under five remained relatively stable between 2016 and 2022, underscoring the persistent and insufficiently addressed burden of pediatric injuries in China (25). Addressing this challenge requires a dual-pronged approach: strengthening legislative and regulatory frameworks for child injury prevention based on established risk factors and empirical evidence, and implementing community-based interventions that promote caregiver education, safe sleep practices, and the reduction of household environmental hazards.

Several limitations should be acknowledged. First, the analysis was based on data from a single province. Although Shaanxi Province shares important demographic, socioeconomic, and health-system characteristics with other less-developed provinces in Northwest China, including a large rural population, substantial within-province disparities, and heterogeneous access to maternal and neonatal care, direct extrapolation of the findings to the broader Northwest Region or to other provinces should be undertaken with caution. Differences in baseline mortality levels, health service capacity, and surveillance coverage may influence both the magnitude and the trends of cause-specific infant mortality. Second, with the development and increasing availability of advanced diagnostic technologies, the accuracy of cause-of-death reporting has likely improved over time, which may introduce classification inconsistencies across different periods. In addition, urban–rural differences in case ascertainment should be considered, as a higher proportion of rural infant deaths occurred outside tertiary medical facilities, which may increase the risk of diagnostic misclassification. Although standardized multi-tier verification procedures were implemented within the surveillance system, some residual differential misclassification cannot be completely excluded. Third, the definition of live birth used in the MCHSS differs from the WHO definition, which records all live births regardless of gestational age or birth weight. This difference may limit direct international comparability of infant mortality rates, although extremely pre-term births accounted for only a very small proportion of live births in the present study. Finally, the MCHSS dataset does not include detailed sociodemographic or healthcare access variables, such as household income, maternal comorbidities, or environmental exposures. The absence of these covariates limited our ability to assess the potential influence of contextual determinants on infant mortality. Future studies incorporating more comprehensive individual and contextual data may provide a deeper understanding of mortality patterns and support more targeted intervention strategies.

In conclusion, infant mortality in Shaanxi Province declined substantially between 2014 and 2023, with parallel reductions observed in urban and rural populations. Cause-specific mortality from congenital anomalies and pneumonia decreased significantly, whereas pre-maturity- and injury-related causes remain important contributors to infant death. Continued strengthening of neonatal care systems, birth defect prevention programs, and injury prevention strategies, particularly in underserved rural areas, will be essential to sustain progress and advance equitable child survival.

## Data Availability

The original contributions presented in the study are included in the article/Supplementary material, further inquiries can be directed to the corresponding author.
