# Supplementary material for: Trends and urban–rural disparities in infant and cause-specific mortality in Shaanxi Province, China, 2014–2023: a population-based study
Source: Front Public Health. 2026 Apr 14;14:1801740. doi: 10.3389/fpubh.2026.1801740 (PMC13121391; doi:10.3389/fpubh.2026.1801740)
Supplement: Supplementary file 1 [file Data_Sheet_1.pdf]

Trends and Urban–Rural Disparities in Infant and Cause-Specific Mortality in Shaanxi Province, China, 2014–2023: A Population-Based Study

Xue Yang*, Baozhu Wang and Juan Zhang

Supplementary Table S1. Distribution of causes of infant death by age at death in Shaanxi Province, 2014–2023. LBW: low birth weight.

Causes of death	< 6 days	7-27 days	≥ 28 days
Congenital heart defects	40 (26.1)	27 (17.6)	86 (56.2)
Birth asphyxia	140 (95.2)	5 (3.4)	2 (1.4)
Preterm/LBW	92 (68.1)	26 (19.3)	17 (12.6)
Pneumonia	24 (23.8)	20 (19.8)	57 (56.4)
Other congenital anomalies	63 (65.6)	11 (11.5)	22 (22.9)
Accidental asphyxia	29 (34.5)	13 (15.5)	42(50.0)
Others	68 (28.6)	34 (14.3)	136 (57.1)
